# Yield of active screening for tuberculosis among asylum seekers in Germany: a systematic review and meta-analysis

**DOI:** 10.2807/1560-7917.ES.2017.22.12.30491

**Published:** 2017-03-23

**Authors:** Kayvan Bozorgmehr, Oliver Razum, Daniel Saure, Brigitte Joggerst, Joachim Szecsenyi, Christian Stock

**Affiliations:** 1 Department of General Practice and Health Services Research, University Hospital Heidelberg, Heidelberg, Germany; 2 Department of Epidemiology and International Public Health, School of Public Health, Bielefeld University, Bielefeld, Germany; 3 Institute of Medical Biometry and Informatics, University of Heidelberg, Heidelberg, Germany; 4 Public health authority, Pforzheim, Germany

**Keywords:** tuberculosis, infection control, public health policy, migrant, migration, asylum seekers, refugees, screening, review

## Abstract

All asylum seekers in Germany undergo upon-entry screening for tuberculosis TB, but comprehensive evidence on the yield is lacking. We compared the national estimates with the international literature in a systematic review and meta-analysis of studies reporting the yield of TB, defined as the fraction of active TB cases detected among asylum seekers screened in Germany upon entry. We searched 11 national and international databases for empirical studies and the internet for grey literature published in English or German without restrictions on publication time. Among 1,253 screened articles, we identified six articles reporting the yield of active TB based on German data, ranging from 0.72 (95% confidence interval (CI): 0.45–1.10) to 6.41 (95% CI: 4.19–9.37) per 1,000 asylum seekers. The pooled estimate across all studies was 3.47 (95% CI: 1.78–5.73; I^2^ = 94.9%; p < 0.0001) per 1,000 asylum seekers. This estimate was in line with international evidence (I^2^ = 0%; p for heterogeneity 0.55). The meta-analysis of available international estimates resulted in a pooled yield of 3.04 (95% CI: 2.24–3.96) per 1,000. This study provides an estimate across several German federal states for the yield of TB screening in asylum seekers. Further research is needed to develop more targeted screening programmes.

## Introduction

Substantial progress has been made in the control of tuberculosis (TB) since the ratification of the Millennium Development Goals, but the disease still remains a major global health problem and a leading cause of death worldwide [1]. Because of increasingly complex forms of migration [2], including migration from high-incidence TB countries and perimigration factors favouring transmission or re-activation of TB, the disease remains a public health concern also for low-incidence countries with notification rates below 10 per 100,000 population [3]. The incidence (not the transmission [4-6]) of TB in many low-incidence countries is driven largely by international migration. The epidemiology in these countries is characterised by the progression of latent TB infection rather than recent transmission, and by a high concentration of cases in vulnerable and hard-to-reach risk groups such as migrants, in particular refugees from high-incidence TB countries [3]. Between 2015 and 2016, the European Union (EU) received more than 1.3 million first-time asylum applicants. Among the top 10 countries of origin of asylum seekers in this period, six countries (Afghanistan, Eritrea, Nigeria, Pakistan, Russia and Ukraine) with TB incidence rates above 50 per 100,000 accounted for more than 25% of the total number of asylum applicants [7].

Immigration medical screening has played a major role in TB control programmes for more than a century [8]. In many low-incidence countries, it is a cornerstone of national TB control programmes [9] and comprises pre-entry, upon-entry and post-entry screening programmes [9,10]. The majority of EU countries [9,11] and member countries of the Organisation for Economic Co-operation and Development (OECD) [12] have mandatory upon-entry TB screening programmes for immigrants, including refugees and asylum seekers. Chest radiography (X-ray) alone or in combination with other screening approaches (such as clinical examination or tuberculin skin test) constituted the most frequently applied measure in 22 of 29 OECD countries to screen for active TB in the year 2010 [12].

Germany is a low-incidence TB country with an incidence rate of 5.6 cases per 100,000 population (4,488 cases were notified in 2014) [13]. Screening for TB in migrants is regulated by national law and restricted to specific migrant groups. According to §62 of the Asylum Law (Asylgesetz, AsylG – formerly: Asylverfahrensgesetz) in combination with §36 of the Infection Protection Act (Infektionsschutzgesetz, IfSG), foreigners (except pregnant women) aged 16 years or older and living in shared accommodation facilities such as reception centres or shelters for asylum seekers must undergo a compulsory chest X-ray examination, primarily to identify active pulmonary tuberculosis. Further measures of upon-entry screening for TB, especially in children or pregnant women, are governed by different policies at the level of the 16 federal states [14].

In 2014, TB incidence in residents with foreign nationalities in Germany was 33.6 cases per 100,000 population, which is 13 times higher than the incidence in German citizens (2.5 cases per 100,000 population) [15]. Between 2001 and 2014, 2.9% of all notified TB cases were identified in the scope of the above legal frameworks among asylum seekers. While the share of TB cases in asylum seekers among all incident TB cases in Germany was 0.8% in 2008, this proportion rose to 10.6% in 2014 [15]. The number of refugees seeking asylum in Germany increased continuously in the same time period [16] and reached 1.1 million in 2015 [17].

Germany has a well-functioning national TB surveillance programme with mandatory reporting since 1934. TB notification data can be stratified by nationality and by ‘reason of the diagnostic measure’. This allows distinguishing between cases identified by passive vs active case finding, e.g. in the scope of (active) upon-entry screening among asylum seekers.

While notification of identified cases is mandatory in the decentralised German health system, there is no legal obligation to document nor to report the number of asylum seekers screened upon entry within the framework of related legal frameworks (AsylG, IfSG). Therefore, incidence rates cannot be calculated routinely for this group, and no information on the yield of TB screening programmes is easily available on national level. This information, however, would be of high importance for evaluating effectiveness and cost-effectiveness and for attempts to prioritise specific high-risk groups. The aim of this study was to synthesise evidence on the yield of entry screening programmes for TB among asylum seekers in Germany, and to compare the estimate with international evidence.

## Methods

### Study design

We performed a systematic review and meta-analysis of the literature reporting the yield of entry screening programmes for TB among asylum seekers in Germany. Yield was defined as the fraction of active TB cases detected among 1,000 asylum seekers screened.

The literature was retrieved in the scope of a broader configurative systematic review [18] aimed at identifying and mapping all empirical studies on health and healthcare among asylum seekers and refugees in Germany [19]. The protocol of the configurative systematic review and evidence-mapping study was registered in an international prospective register of systematic reviews (PROSPERO 2014:CRD42014013043) and published in a peer-reviewed journal before starting the review [19]. The evidence map and synthesis generated by the configurative review laid the foundation for this aggregative review. This type of review seeks to add up and average (homogenous) empirical observations in order to make empirical statements within narrower predefined concepts to inform decisions. Aggregative reviews can follow configurative ones, which aim to provide concepts and patterns among heterogeneous and more complex fields [18].

### Review question and outcome

The question for this systematic review and meta-analysis was formulated as follows: What is the yield of upon-entry screening for TB among asylum seekers in Germany? The primary outcome was the yield of TB among asylum seekers screened in the scope of active screening programmes (according to §62 AsylG in combination with §36 IfSG).

### Search strategy

A three-tiered search strategy was applied:

1. We searched 11 bibliographical databases for indexed articles: PubMed/MEDLINE, ISI Web of Science, International Bibliography of Social Sciences (IBSS), Sociological Abstracts, Social Science Citation Index (SSCI), Worldwide Political Science Abstracts (WPolScA), Cumulative Index to Nursing and Allied Health Literature (CINAHL), Sowiport, Applied Social Sciences Index and Abstracts (ASSIA), Medpilot, German National Library (DNB). In addition, we searched the Internet via Google in order to identify grey literature. The searches were performed in August and September 2014 (Web of Science, Medpilot: 22 Aug 2014; SSCI, ASSIA: 24 Aug 20e Aug 2114; PubMed, IBSS, Sociological Abstracts, WPolScA: 9 Sep 2014; CINAHL, DNB: 30 Sep 2014; Google: 2 Sep 2014).

2. We reviewed the reference lists of included articles to retrieve further indexed articles.

3. We contacted 47 experts from 31 organisations inquiring for grey literature.

4. We updated the database search in PubMed/MEDLINE for the period from September 2014 to 26 March 2016 to ensure that articles published since the initial search were considered.

No time limitation was set for the searches. For the full text screening, we excluded studies published before 1990 due to their historical character, since major legal regulations governing screening for TB in asylum seekers (AsylVfG) were not introduced in national law before the 1990s.

#### Search terms

Search terms were tailored to the broader scope of the configurative systematic review and evidence-mapping study and did not include terms specific for tuberculosis screening [19]. The search terms ((refugee* OR asylum*) AND (health* OR access OR utilisation) AND german*) were used for international databases; the terms (Flüchtling OR asyl* AND gesundheit*) for German databases. The search in databases included titles, abstracts and keywords, without any restriction regarding time period or language. For the Internet search, different search term combinations were used as documented in the review protocol [19].

### Inclusion and exclusion criteria

#### General eligibility

Articles fulfilling all of the following criteria were eligible for inclusion in the broader evidence-mapping study: (i) empirical articles (i.e. quantitative or qualitative primary studies, as well as reviews of empirical studies), (ii) articles focusing on asylum seekers and refugees in Germany as a distinct study population, (iii) articles reporting on any parameter of health or healthcare provision as outcomes and (iv) articles published in German or English.

The specific type of outcome (e.g. a specific disease or condition) was not defined as a criterion for inclusion or exclusion into the configurative review and evidence-mapping study.

Exclusion criteria for the configurative review were unclear study populations (e.g. migrants of unknown status or lack of stratified results for asylum seekers/refugees as part of general migrant populations) and undocumented migrants, ethnic German resettlers (*Aussiedler*), persons internally displaced in the context of World War II or refugees from the German Democratic Republic as the study population. We also excluded non-empirical literature (commentaries, working papers, journalistic interviews, policy reports, books, conference transcripts or congress abstracts without available full texts).

Studies were excluded and assigned to a residual category not considered for the evidence mapping if they reported findings of international studies without stratified data for Germany or turned out to be secondary literature not exclusively based on empirical material.

#### Tuberculosis-specific eligibility

Articles meeting the above general criteria were eligible for inclusion in this review if: (i) they reported the number of active TB cases detected in the scope of entry screening programmes and (ii) provided accurate information on denominators of the screened population of asylum seekers.

Articles retrieved by the updated database search were screened using the general criteria (i), (ii) and (iv) together with the TB-specific eligibility criteria in one step, i.e. without the intermediate step of applying the general eligibility criterion (iii).

### Screening and study selection

All retrieved references (titles and abstracts) were screened independently by two reviewers of the initial review team [19,20]. The full texts of articles included after abstract/title screening were again screened independently by the same reviewers. Any discrepant judgements on eligibility were discussed in consensus meetings among at least three members of the initial review team [19,20] and articles were included or excluded after reaching mutual agreement. References retrieved in the updated search (titles, abstracts and full texts) were screened by the first author (KB).

#### Effectiveness of the search strategy and sensitivity analysis

The effectiveness of the search strategy of the configurative review was assessed by calculating its specificity and sensitivity. Specificity was assessed by the proportion of eligible articles among all search results. Sensitivity was calculated as the proportion of eligible articles identified by the search among all truly eligible articles (true positives and false negatives) using a test set of articles a priori defined and listed by the authors before starting the review [20]. In order to rule out the possibility of a selection bias for the aggregative review, we performed a sensitivity analysis: the updated search in Pubmed/MEDLINE (Sep 2014–26 Mar 2016) was repeated with extended search terms including terms for migrants derived from medical subject headings (MeSH). The final Boolean operator for the updated search with extended search terms was: (refugee* OR asylum* OR foreign* OR immigrant* OR migrant* OR emigrant*) AND (health* OR access OR utilisation) AND german*. Applying the same inclusion/exclusion criteria, we assessed whether this extended search yielded any further eligible articles that were not previously identified.

#### Data extraction

We systematically extracted generic information on included articles (authors, year of publication, type of publication and funding sources) and the following content-specific information: research questions, study context/setting, study period, study populations and socio-demographic variables (age, sex and country of origin), sampling strategy, total number of asylum seekers, number of asylum seekers undergoing upon-entry screening, number of active TB cases identified, case definitions and diagnostic methods as reported, limitations as reported and statements on generalisability with respect to the outcome of the review.

#### Critical appraisal

Studies were categorised according to the Levels of Evidence (LoE) of the Oxford Centre for Evidence-Based Medicine based on the study type of the primary article [21]. Additional limitations beyond those reported in the primary articles were identified by the reviewers and documented in the extraction sheets. We assessed the external validity of studies on the basis of reported limitations, reported external validity and additional limitations identified by the reviewers. We also categorised the generalisability of findings with respect to the local, regional or supraregional level. In this context, ‘local’ referred to the generalisability of findings to the population of one single accommodation, ‘regional’ referred to the generalisability to the population of one city or region and ‘supraregional’ referred to the generalisability across federal states.

### Statistical analysis and evidence synthesis

We calculated the coverage of screening programmes as the proportion of asylum seekers undergoing screening among total numbers of asylum seekers. The yield of TB screening programmes was calculated as the fraction of active TB cases detected among the number of asylum seekers undergoing screening (expressed as cases per 1,000 persons). Authors of primary studies were contacted for further information if the reported data was not sufficient to calculate the yield.

In a random-effects meta-analysis, the yield was synthesised across studies and pooled estimates along with corresponding 95% confidence intervals (CI) were calculated, weighting each study by its inverse variance, applying the DerSimonian-Laird estimator for between-study variance and the arcsine transformation to calculate the overall yield. As considerable clinical heterogeneity was expected, a random-effects rather than a fixed-effect model was applied. Sensitivity analyses were performed to assess the influence of potential over-reporting of active TB cases in primary studies with imprecise case definitions. In order to estimate the numbers of asylum seekers that would need to be screened to detect one case of TB, the pooled estimates of the yield and corresponding confidence limits were inverted. Results of a meta-analysis of the yield of TB screening among asylum seekers with no restriction of the host country (but not including studies from Germany) performed by Arshad et al. were used for comparison with international studies [22]. An updated pooled estimate combining the individual studies included in this review and in Arshad et al. [22] was calculated using the same approach as described above. Minor differences compared with the results reported by Arshad et al. were due to a slightly different meta-analytical approach, e.g. in the computation of confidence intervals. The meta-analyses were performed in the R language and environment for statistical computing (Version 3.3, The R Foundation for Statistical Computing) using the R-package ‘meta’ (Version 4.5–0).

## Results

**Figure 1:**
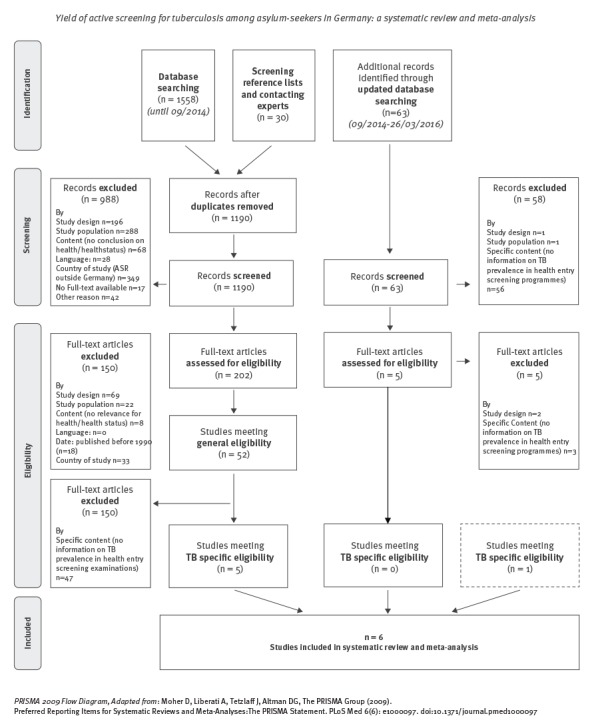
Flowchart of the review process, tuberculosis screening among asylum seekers in Germany

ASR: asylum seekers or refugees; TB: tuberculosis.

After removal of 398 duplicates, the search in databases and reference lists and the queries among experts yielded 1,190 hits. Another 63 hits were obtained by updating the search in PubMed/MEDLINE, so that a total of 1,253 articles were screened (Figure 1).

Dotted line: grey literature.

PRISMA 2009 flow diagram, adapted from: [45].

Of these, we excluded 1,046 (83%) after screening of titles and abstracts. The full texts of the remaining 207 articles (of which 12 had some reference to TB) were checked against the general and specific inclusion criteria. This led to the exclusion of another 202 articles so that a total of five studies (0.4% of all hits) were included in the systematic review and meta-analysis via formal searches [23-27]. A relevant grey-literature article published in 2015 after the initial search had been conducted was included while writing up the report [28], so that in total six articles were included in the final analysis.

The included studies [23-28] reported the yield of screening for tuberculosis among asylum seekers upon-entry in three large federal states [25,26,28], two of the smallest federal states [23,27] and in the city of Munich [24]. No study reported findings across more than one federal state (Table).

**Table:** Characteristics and extracted details of included studies on tuberculosis screening in asylum seekers in Germany (n = 6)

Reference	Type of publication	Study objective(s)	Study design	Setting/context of study	Year of data collection	Coverage (%)	Country/countries of origin of the screened population	Men (%)	Age groups (in years): % or n	Sampling strategy	Stratification	Diagnostic methods	Case definitions of TB	Main limitations (as reported)	Additional (main) limitations identified by review-team	Funding sources/ conflicts of interest	Level of evidence
Diel et al. (2004) [23]	International journal, externally peer-reviewed	To study the characteristics of TB in foreign-born individuals living in Hamburg	Prospective, population-based molecular-epidemiological study	State of Hamburg	1997–2002	95.5	Afghanistan: 48.6%, Turkey: 7.5%, Iran: 6.5%, Burkina Faso: 4.9%, ex-Yugoslavia^a^: 4.3%, Sierra Leone: 4.1%, Russia: 2.7%, Guinea: 1.7%, Togo: 1.5%, Egypt: 1.3%, Other: 17.3%	48.1	No age groups given (NA)	All patients with culture- confirmed TB reported to the seven district public health departments in Hamburg; this includes 12,176 of 12,751 asylum seekers in Hamburg who were screened at entry	None	General health examination; chest X-ray; tuberculin skin testing; Bacterial strains and drug susceptibility testing; IS6110 DNA fingerprint analysis	Extrapulmonary TB (defined as disease with no evidence of lung involvement) and pulmonary TB (sputum-positive or culture-positive)	Limitation of the study period to 5.5 years resulted in an underestimation of the real trans- mission rate between foreign-born and German-born individuals	Study limited to Hamburg	Robert Koch Institute, Berlin, Germany, and the EU Concerted Action project “New Generation Genetic Markers and Techniques for the Epidemiology and Control of Tuberculosis”	1b
Dreweck et al. (2013) [24]	National journal, externally peer-reviewed	NA (implicitly: to provide a descriptive epidemiological report on TB epidemiology in Munich)	Cross-sectional descriptive study	City of Munich	2011, 2012	NA	NA	NA	NA	TB screening of all asylum seekers	None	X-ray	No case definitions provided	Not reported	(i) Denominator not reported precisely, detailed denominator provided after contacting authors; denominators for other years completely missing (ii) No information on countries of origin, age or sex	None reported	3b
Joggerst and Käßmann (2013) [25]	Supplement/congress abstract in national journal (further information provided in the form of a detailed poster)	To analyse the results of the health entry examination at the reception centre Karlsruhe over a period of 10 years	Prospective population-based study, cross-sectional time-series	Main reception centre of the State of Baden-Wurttemberg	2002–11	NA	Irak: 16.1%, Turkey: 9.7%, Serbia: 6.7%, Pakistan: 5.4%, Iran: 5.1%, Cameroon: 4.7%, Afghanistan: 4.7%, Nigeria: 4.4%, Russia: 4.4%, China: 4.4%, Sri Lanka: 4%, India: 3.8%	72.3	0–10: 8.5%, 11–20: 17.8%, 21–30: 43.8%, 31–40: 20.3%, 41–50: 5.7%, 51–60: 1.9%, 61–70: 1.0%,	Screening of all asylum seekers allocated to the State of Baden-Wurttemberg	By country of origin, age, sex	X-ray for asylum seekers ≥ 16 years; tuberculin test < 16 years	No case definitions provided	Not reported	Abstract; assessment of observed vs expected cases only descriptive and based on a single year of WHO data (2011)	None reported	1b
Kesseler et al. (1995) [26]	National journal, externally peer-reviewed	To evaluate the prevalence of active and latent TB in asylum seekers	Prospective observational study	Nine public health offices in North Rhine-Westphalia	1992–94	100	Europe: 70%, Yugoslavia^a^: 42%, Romania: 14%, Bulgaria: 3%, Turkey: 7%, Africa: 13%, Asia: 7%	72	Range: 1–89. 0–10: 86, 11–20: 716, 21–30: 1,953, 31–40: 875, 41–50: 261, 51–60: 113, > 61: 53	All asylum seekers in study area (9 Health Offices in NRW) from June 1992 to January 1994	None	Chest X-ray, tuberculin test	Active pulmonary TB (culture-positive, smear-positive or smear-negative)	Not reported	No further characteristics provided/assessed in stratified analysis which could affect TB prevalence identified by screening	None reported	1b
Michels and Bartz (2015) [28]	National journal, in-house peer-reviewed	To report the results of the TB screening among asylum seekers in the scope of the health entry examination at the reception centre Trier	Prospective population-based study	Main reception centre of the State of Rhineland-Palatinate	2001–14	100	Reported only for a subsample of the year 2014 (n = 10,528): Syria: 2,835, six cases, 212/100,000; Kosovo^b^: 1,130, four cases, 354/100,000 Serbia: 1,057, six cases, 568/100,000; Eritrea: 898, nine cases, 1,002/100,000 Albania: 660, one case, 152/100,000; Somalia: 540, 11 cases, 2,037/100,000 No countries reported: 3,408, 10 cases	NA	NA	Screening of all asylum seekers allocated to the State of Rhineland-Palatinate	By country (only for a subsample of the year 2014)	Chest X-ray (adults) tuberculin test (children/adolescents < 16 years, pregnant women); in single cases additionally IGRA, sputum diagnostic, further serological tests (not further specified), chest CT scan	22 cases: culture/sputum-positive 21 cases: culture/sputum-negative four cases: unclear	Not reported	Age and sex of screened population and of cases not reported; unclear how many cases were identified by which diagnostic method; countries reported only for a subset of the 2014 population; comparison with WHO prevalence rates only descriptive and only for 2014	None reported	1b
Mohammadzadeh (1995) [27]	National journal, externally peer-reviewed	To evaluate the Initial Health Examination Programme in Bremen	Retrospective study of medical records	Initial Health Examination Programme in Bremen	June 1993-June 1994	59.9	ex-Yugoslavia^a^, Romania, Commonwealth of Independent States, Algeria, Turkey, Sierra Leone, Liberia, Togo, Iran, Bulgaria (no further details reported)	80.4	NA	All asylum seekers who sought care in the programme	Adults vs children (categories not further specified)	Methods of diagnosis not specified, chart review of medical records/routine data collected in the programme	Reported cases include history of TB, suspected TB, extrapulmonary (nodal) TB, and pulmonary TB (all types: not further specified)	NA	No descriptive information on study population; missing information on data collection/diagnostic methods	Programme conducted by Health Office Bremen	2c

CT: computed tomography; EU: European Union; IGRA: interferon gamma release assay; NA: not available. TB: tuberculosis; WHO: World Health Organization.

^a^ Country name listed as per original publication.

^b^ Kosovo under UN Security Council Resolution 1244 in 1996.

### Characteristics and quality of included studies

The included studies were very heterogeneous with respect to primary objectives, study design and type of publication. The primary objective of three studies was to assess TB prevalence in asylum seekers in the scope of screening programmes [25,26,28]. The remaining studies pursued other primary objectives and reported the yields of screening programmes as secondary findings [23,24,27].

Further heterogeneity was found in study designs: four articles were prospective observational studies (LoE 1b), one was a cross-sectional (LoE 3b) [24] one a retrospective medical records study (LoE 2c) [27].

All reports were published in peer-reviewed journals (including those with in-house peer review), but only one was published in English and in an international journal [23]. The reports included a published congress abstract which we included since additional information (in form of a poster) and access to the primary data were granted by the authors so that sufficient information was at hand to ensure eligibility [25].

The findings of five studies were regionally generalisable at the level of the respective federal states [23,25-28]. None of the studies made formal comparisons with the characteristics of asylum seeker populations at national level, so that an assessment of the representativeness of samples beyond regional boundaries was not possible. Only one study reported study limitations in detail [23]. Limitations of the primary reports identified by the review team are provided in the Table.

Case definitions ranged from none [24] or poorly reported ones [27] to clear definitions of identified TB cases [23,26,28]. The chest X-ray as a diagnostic method to screen for active TB cases was clearly reported by all but one study [27]. Studies reporting more than one diagnostic method did not report the number of cases identified by each method [28]. Three studies reported stratified results [25,27,28], but stratification was incomplete and rudimentary in all but one [25]. One study provided detailed stratification of results only for migrants, but not for the sub-group of asylum seekers [23].

### Sample sizes and yield of screening programmes

**Figure 2:**
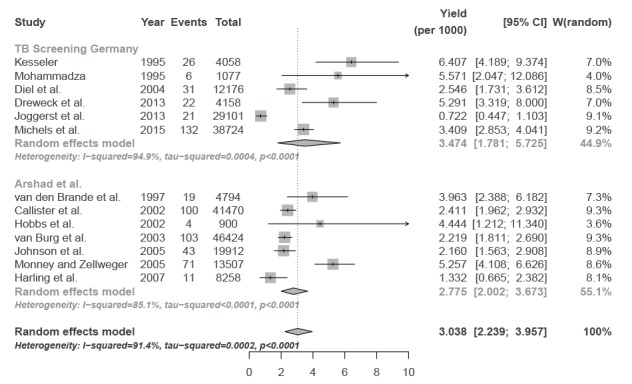
Forest plots of the yield of tuberculosis cases in screening studies in Germany (n = 6 studies) and in component studies included in an international review (n = 7 studies), as well as joint pooled estimate

CI: confidence interval; TB: tuberculosis; W (random): weight of study in random effects model.

The sample sizes of screened asylum seekers ranged from n = 1,077 (smallest study) to n = 38,724 (largest study), the mean and median numbers of screened asylum seekers were n = 14,882 and n = 8,167, respectively. The included studies comprised a total of 89,294 asylum seekers (Figure 2, upper part).

Component studies from the international review are taken from Arshad et al. [22].

The number of reported TB cases identified by upon-entry screening ranged from six to 132 (mean: 24; median: 39.7). The yield of screening programmes in primary studies ranged from 0.72 (95% CI: 0.45–1.10) [25] to 6.41 (95% CI: 4.19–9.37) [26] cases per 1,000 asylum seekers. The pooled estimate for the yield of TB screening programmes across all studies was 3.47 (95% CI: 1.78–5.73) cases per 1,000 asylum seekers (Figure 2, upper part). This corresponded to 288 (95% CI: 175–561) asylum seekers that would need to be screened to detect one case of TB. The meta-analysis revealed substantial statistical heterogeneity among the studies (I^2^ = 94.9%; test for heterogeneity: p < 0.0001).

In a sensitivity analysis, we calculated a conservative estimate by excluding four TB cases (suspected cases and histories of TB) reported by Mohammadzadeh [27]. The conservative pooled estimate for the yield of upon-entry screening across all studies was 12.1% lower (3.05 (95% CI: 1.50–5.14) per 1,000 asylum seekers) than the yield of the non-conservative estimate (3.45 (95% CI: 1.78–5.73) per 1,000 asylum seekers), which would correspond to 327 (95% CI: 194–667) asylum seekers to be screened in order to detect of one case of TB.

The pooled point estimate of the yield of TB identified by screening programmes in the German studies was slightly higher than the pooled point estimate of 2.70 (95% CI: 1.98–3.42) per 1,000 asylum seekers reported by Arshad et al. who performed a meta-analysis of seven international primary studies with a total of 351 TB cases identified by screening of 135,265 asylum seekers [22]. In a re-analysis of the data included in Arshad et al., using the same methods as applied above for the German data, we obtained a point estimate of 2.77 (95% CI: 2.05–3.75) per 1,000 asylum seekers, as shown in Figure 2 (lower part). The meta-analytic comparison of the pooled estimate of the yields reported by German studies and that of international studies [22] exhibited no statistical heterogeneity (I^2^ = 0%; test for heterogeneity: p = 0.55; data not shown). The pooled overall yield was 3.04 (95% CI: 2.24–3.67), as shown in Figure 2, which corresponded to 329 (95% CI: 253–447) asylum seekers that would need to be screened to detect one case of TB.

### Effectiveness of the search strategy and sensitivity analysis

The search strategy for the configurative review identified 52 relevant articles from a total of 1,190 hits. This corresponded to a specificity of 4.4%, which was to be expected when applying such a broad search strategy. The sensitivity of the search strategy was 98.1% when based on the articles of the test set [19] including grey literature and 100% when based on the articles from peer-reviewed journals.

The sensitivity analysis using extended search terms related to migration yielded 295 hits in the updated search (compared with 63 hits when using specific search terms for the migrant population in question, Figure 1). Of these, 288 were excluded for study design (n = 117), for study population, i.e. lack of focus on asylum seekers or refugees (n = 56), for specific content, i.e. no relation to TB or no information on TB yield in health entry screening programmes (n = 93) or for country of study (n = 22). The remaining seven articles [29-35] were assessed in full text for eligibility. These were excluded for study design (n = 3), for lack of reference to TB or TB yield in screening programmes (n = 3), or for country (n = 1), so that no additional studies were included in the systematic review after broadening the search terms to include a reference to overall migrant groups.

## Discussion

The yield of upon-entry screening programmes for TB in asylum seekers as assessed by this systematic review and meta-analysis of studies in Germany was 3.47 (95% CI: 1.78–5.73) per 1,000 asylum seekers. This corresponds to a number needed to screen (NNS) of 288 (95% CI: 175–561) asylum seekers to identify one case of TB. The pooled estimate derived from the meta-analysis of German studies concurs with international findings on the yield of active TB screening programmes for asylum seekers upon entry [22]. The joint yield of German and international studies was 3.04 (95% CI: 2.24–3.67), corresponding to a slightly higher NNS of 329 (95% CI: 253–447) to identify one case of TB in asylum seekers. The review by Arshad et al. considered studies performing both radiological and microbiological tests to identify cases of active tuberculosis [22], so that the applied screening strategies are comparable. According to a systematic review performed in 2013 by the World Health Organization (WHO), the overall median NNS of immigration screening (considering mixed migrant groups in the scope of immigrant, border and refugee screening) was 156 (95% CI: 66–320) [36]. The weighted mean NNS based on 3,429,573 individuals screened in 38 studies was 108 (95% CI: 6–1,630) [36]. The overall NNS in our study was higher, corresponding to a lower yield of screening. This may be explained by differences in migrant groups, migration routes and countries of origin. Other reviews comparing different types of screening (pre-, upon- or post-entry screening) for TB in migrants in low-incidence TB countries report high variations in the yield of screening [37]. This may explain the different conclusions of health economic evaluations regarding the cost-effectiveness of screening for active TB [38]. Further health-economic analyses and rigorous studies on the effectiveness of TB screening are thus needed to assess the impact on both transmission of TB and individual health outcomes [38,39].

Similar to the primary studies identified by Arshad et al. only few primary studies in our review reported yields stratified by age [25,27], sex [25] or country of origin [25,28], which may partly be explained by low case numbers limiting the possibility of reporting across multiple strata. Important post-migration factors such as median length of stay in the host country and characteristics of the accommodation were not reported either by the primary studies in our review. It is known that the underlying incidence of TB in the countries of origin affects the yield of screening approaches in different settings [36,40]. Better reporting of country-stratified yields may therefore help to prioritise special risk groups among the heterogeneous population of asylum seekers. Two studies [25,28] additionally compared the TB yields by country of origin descriptively with the prevalence rates of asylum seekers’ countries of origin reported by the WHO. Michels and Bartz [28] reported much higher yields among a subsample of asylum seekers originating mostly from high-prevalence countries in the year 2014 than could be expected based on WHO prevalence rates for the respective countries of origin (Albania, Eritrea, Serbia, Somalia and Syria). Joggerst and Käßmann [25] also found more cases than expected for some countries (Turkey and countries within the area of the former Republic of Yugoslavia), but reported fewer cases than expected for others (Afghanistan, Iraq, Liberia and Pakistan). They hypothesised that two different phenomena co-occur among asylum seekers: a ‘healthcare-seeking migration’ from countries that are geographically closer (implying that persons with TB have a higher probability of migrating) and a ‘healthy migrant effect’ for geographically more distant countries (implying that persons with TB have a lower probability of migrating).

Further factors beyond selection effects, such as transmission and re-activations during the flight, as well as post-migration factors such as accommodation, may also explain the increased yields.

### Strengths and limitations

The major strength of this systematic review is the comprehensive search for and meta-analysis of studies on the yield of TB screening programmes in asylum seekers in Germany. This is the only migrant group which systematically undergoes active screening for TB. We generated the first estimate of yields of active screening for TB beyond boundaries of single federal states. All studies but one were published in German, which may be the reason why they were not included in the review by Arshad et al. [22]. We are aware of only one international systematic review [9] that included two studies from Germany [23,26]. Our study provides evidence accessible to an international community on the effectiveness of screening programmes in one of the largest recipient countries for asylum seekers in Europe.

Our analysis is, however, limited by the heterogeneity in study characteristics and also in study results (estimates of the yield of TB) across primary studies. This includes poorly reported case definitions and heterogeneous diagnostic methods (except for the chest X-ray). Because of the limited socio-demographic information provided in primary reports and the lack of stratified findings and numbers of events it was not possible to track the reason for this heterogeneity. A likely explanation is that we pooled estimates from different waves of asylum seekers which differed with respect to the major countries of origin, the reasons for migration and the conditions during migration and reception.

Our search strategy was broad and unspecific, but highly sensitive. We therefore rule out the possibility of a selection bias as explanation for the small number of identified studies. We identified all relevant articles on the group of asylum seekers and on migrant groups labelled as ‘refugees’ in Germany and included those with a reference to active screening for TB in the aggregative review. We excluded studies on other specified migrant populations (e.g. undocumented migrants), but studies reporting populations of ‘general migrants’ or ‘immigrants’ in the abstract or title without any further specifications were not excluded at the stage of screening the abstracts and titles and included in the full-text screening. They were only excluded if it became clear at the stage of full-text screening that the study population, i.e. asylum seekers, was not addressed or not specifically distinguished in the results section.

Although the initial search terms did not include terms related to migration in general, our search strategy identified relevant studies that used the term ‘immigrant’ in the title (e.g. [23]), but reported the study population of concern for our review (asylum seekers) in the abstract or as part of the keywords. Our search terms were maximally broad with respect to the outcomes (health and healthcare), and broadening the population to include general ‘migrants’ in the searches would have decreased specificity even further to unacceptably low levels, increasing the work load. The numbers of hits yielded by the updated search with extended terms was about five times (4.7) higher than the number of hits yielded by the search with more specific search terms. However, no additional studies were identified despite the broader search. Firstly, there is no TB screening for regular immigrants or general migrants in Germany. Active screening for TB is performed exclusively among asylum seekers, so broadening the population to general migrants would not yield more relevant articles in the German context.

### Recommendations for further research

More research is necessary to assess the yield of screening programmes for TB depending on country of origin. This is not a purely academic issue, but has highly important practical implications. Screening for TB among asylum seekers upon entry in times of high immigration constitutes a substantial challenge for public health authorities [14]. The limited evidence provided by country-stratified analysis shows the importance of a targeted approach such as prioritising high-risk groups when time and personnel resources are limited, especially during periods of large-scale immigration of asylum seekers.

However, targeted screening among immigrants was performed in only six of 25 OECD countries in 2010 using thresholds based on the TB incidence in their country of origin. Incidence thresholds at which screening was initiated ranged from more than 15 to more than 100 cases per 100,000 population [12].

Another question of public health relevance is to establish the effectiveness of screening programmes beyond yields. Timeliness of case detection and treatment outcomes are highly important, but evidence on these aspects in asylum seekers is rare. National [20] and international systematic reviews [41] identified only one study analysing TB treatment outcomes in asylum seekers in Germany [42]. This study shows that treatment failure is disproportionately higher among asylum seekers than among the native population [42].

Furthermore, data on cases with drug resistance or multidrug resistance would be necessary to fully understand the risk posed by specific subgroups. As cases of resistant or multidrug-resistant TB are far more dangerous, screening in subgroups with a known high risk of resistance needs to be more extensive, even if absolute case numbers are low.

There is no or no comprehensive screening for children among refugees [43]. National TB screening protocols (AsylG, IfSG) do not address the issue of TB screening for children younger than 15 years. In 2013, TB incidence in Germany in children was 1,6 per 100,000; 35% of cases were foreign-born children [44]. The individual risk of children to develop serious and generalised infections is high, and there is no evidence for an age limit at which there is no risk for transmissions [44]. The tuberculin skin test (TST) is recommended for screening of asylum-seeking children under the age of 5 years, and TST or interferon-gamma release assay are recommended for screening of children aged 5–14 years [44]. However, TB screening policies at federal state level handle this issue very heterogeneously. Because reporting is not stratified by age and the links between diagnostic methods and identified cases are not clear, we could not estimate the TB yield in asylum-seeking children based on the included studies.

Further studies with more detailed information on case finding rates by specific characteristics of the heterogeneous population of asylum seekers are necessary to move from retrospective evaluations of the effectiveness of screening programmes to a prospective prediction of TB risk (by age, sex, country of origin and other characteristics) among newly arriving asylum seekers. Given the unexpectedly high yields in some subgroups, it would also be important to establish factors during migration and initial accommodation which may lead to higher transmission rates or re-activation of latent TB infections, and to prioritise targeted screening in situations of high workload or limited resources.
